# When simulation becomes physical: vicarious symptoms in standardized patients during osces

**DOI:** 10.1186/s12909-025-07950-w

**Published:** 2025-10-28

**Authors:** Alexis Rybak, Maxens Decavele, Jessica Taytard, Manon Allaire, Nada Sabourdin, Laure Serresse, Nadia Nathan, Yanis Tamzali, Marie-Christine Renaud, Alain Carrié, Jean-Philippe Foy, Antoine Monsel, Hugo Bottemanne

**Affiliations:** 1 Department of Pediatrics, Department Woman-Mother-Child, Lausanne University Hospital (Centre Hospitalier Universitaire Vaudois), University of Lausanne, Lausanne, Switzerland; 2 OSCE Research Group, Sorbonne University, Paris, France; 3 Neurophysiologie Respiratoire Expérimentale et Clinique, INSERM UMRS 1158, Sorbonne University, Paris, France; 4 Service de Médecine Intensive et Réanimation (Département R3S), La Pitié- Salpêtrière Hospital, Assistance Publique-Hôpitaux de Paris, Sorbonne University, Paris, France; 5 Pediatric Pulmonology Department and Reference Center for Rare Lung Diseases RespiRare, Armand Trousseau Hospital, Assistance Publique- Hôpitaux de Paris, Sorbonne University, Paris, France; 6 Department of Hepato-gastroenterology, Assistance Publique-Hôpitaux de Paris, Hôpital Pitié-Salpêtrière, Sorbonne University, Paris, France; 7 Team Proliferation, Stress and Liver Physiopathology, INSERM UMRS 1138, Centre de Recherche des Cordeliers, Paris, France; 8 Department of Anesthesiology and Intensive Care, DMU DREAM, Hospital La Pitié Salpêtrière, Assistance Publique-Hôpitaux de Paris, Sorbonne University, Paris, France; 9 GRC 29 Groupe de Recherche Clinique en Anesthésie Réanimation médecine Périopératoire (ARPE), Assistance Publique-Hôpitaux de Paris, Sorbonne University, Paris, France; 10 Pharmacologie et Evaluation des Thérapeutiques chez L’enfant et la Femme Enceinte, INSERM UMR-S 1343, Paris Cité University, Paris, France; 11 Accompagnement et Soins de Support, Equipe Soins Palliatif, Assistance Publique-Hôpitaux de Paris, Sorbonne University, Hôpitaux Charles-Foix, Pitié-Salpêtrière, Rothschild, Tenon, Trousseau/La Roche- Guyon, Paris, France; 12 Childhood Genetic Diseases Laboratory, INSERM UMRS 933, Armand Trousseau Hospital, Assistance Publique-Hôpitaux de Paris, Sorbonne University, Paris, France; 13 Medical and Surgical Department of Kidney Transplantation, Department of Infectious and Tropical Diseases, Assistance publique-Hôpitaux de Paris, La Pitié-Salpêtrière Hospital, Sorbonne University, Paris, France; 14 Service de Biochimie Endocrinienne et Oncologique, Assistance publique-Hôpitaux de Paris, La Pitié-Salpêtrière Hospital, Sorbonne University, Paris, France; 15 Unité de recherche sur les maladies cardiovasculaires et métaboliques, INSERM UMRS 1166, Sorbonne University, Paris, France; 16 Department of Maxillo-Facial Surgery, Assistance-Publique Hôpitaux de Paris, La Pitié-Salpêtrière Hospital, Sorbonne University, Paris, France; 17 Centre de Recherche de Saint-Antoine, Team Cancer Biology and Therapeutics, INSERM UMRS 938, Sorbonne University, Paris, France; 18 Multidisciplinary Intensive Care Unit, Department of Anesthesiology and Critical Care, La Pitié-Salpêtrière Hospital, Assistance-Publique Hôpitaux de Paris, Sorbonne University, Paris, France; 19 Immunology-Immunopathology-Immunotherapy (I3), INSERM UMR-S 959, Institut National de La Santé et de La Recherche Médicale, Paris, France; 20 Biotherapy (CIC-BTi) and Inflammation-Immunopathology-Biotherapy Department (DHU i2B), La Pitié-Salpêtrière Hospital, Assistance Publique- Hôpitaux de Paris, Paris, France; 21 Department of Psychiatry, Mood Center Paris Saclay, DMU Neurosciences, Bicêtre Hospital, Assistance Publique-Hôpitaux de Paris, Paris-Saclay University, Kremlin-Bicêtre, France; 22 CESP (Centre de Recherche en Epidémiologie et Santé des Populations), MOODS Team, INSERM 1018, Paris-Saclay University, Kremlin- Bicêtre, France

**Keywords:** Objective structured clinical examinations, Standardized patient, Vicarious symptoms, Illness attitudes scale, Multidimensional assessment of interoceptive awareness, Three-domain interoceptive sensations questionnaire

## Abstract

**Background:**

Objective structured clinical examinations (OSCEs) are a cornerstone of undergraduate medical student assessment evaluating encounters between students and simulated patients (SPs). SPs are at risk of developing non-specific symptoms such as stress and anxiety. However, data on physical sensations related to their role, vicarious symptoms (VSs), are limited. We sought to measure the prevalence of VSs among SPs and identify factors among socio-demographic characteristics and psychometric scales associated with their presence.

**Methods:**

This was a prospective single-center cohort study in a large French medical University during three OSCE exams. New VSs and their intensity using 0-100 numerical rate scale were assessed in SPs at the end of the examination day and seven days later using electronical survey. Health anxiety (excessive concern about illness) was measured before the examination using IAS scale. Interoceptive sensitivity (awareness of bodily signals) was also measured before the examination using MAIA-2 and THISQ scales. We performed a multinomial logistic regression to identify characteristics associated with the appearance of VSs.

**Results:**

Among the 428 SPs participating to the OSCEs examens, data from 244 SPs were analyzed. On the day of the OSCE, 12% of participants reported VSs (median intensity of 30, interquartile range 20–50). During the following week, 11% experienced similar symptoms (intensity 50, 30–60). Overall, 20% of SPs reported VSs either during the day of OSCEs or during the following week. Personal experience of a similar condition as the one played (adjusted odds ratio [aOR] 3.07, 95% confidence interval 1.33–7.07, *p* = 0.008) and higher IAS scores (aOR 1.03 per point, 1.01–1.05, *p* = 0.03) were associated with VSs occurrence. MAIA-2 and THISQ scores were not associated with the presence of these symptoms using multivariate analysis.

**Discussion:**

VSs are frequent among SPs particularly when the role is similar to a personal experience and in SP with higher IAS scores. The findings may inform on the selection and the preparation of SPs for OSCEs while shedding light on the influence of symptom portrayal on bodily experiences and its potential influence on the quality of the SPs acting across students.

**Trial registration:**

Not applicable.

**Supplementary Information:**

The online version contains supplementary material available at 10.1186/s12909-025-07950-w.

## Background

Objective structured clinical examinations (OSCEs) are a recognized and effective method to assess clinical skills of undergraduate medical students [1, 2]. Students’ competences are assessed in simulated doctor-patient interviews, that relies on simulated patients (SPs). SPs are recruited and trained to portray clinical situation or symptoms. Designed to be as objective as possible, and to ensure equity between students, OSCEs require high level of reliability and consistency along the examination day from SPs.

Negative impact on SPs induced by exposure to portrayed medical situations during OSCEs has been previously described. Previous research in medical education has shown that SPs frequently experienced non-specific symptoms such as stress and anxiety while they struggled with transitioning into their assigned roles and felt dissatisfied with their perceived performance [3–7]. However, investigations into the physical symptoms experienced by SPs induced by repeatedly portraying specific clinical conditions, or vicarious symptoms (VSs), remain underexplored. The feeling of these unpleasant could have negative emotional consequences on SPs, altering their acting performance, and subsequent equity between students across the examination session.

Functional or somatoform disorders are defined as physical symptoms appearing in a subject in the absence of lesions or defined organic causes. They are common in general population [8, 9]. In the context of psychosomatic medicine, there is a research interest for the potential relation between functional disorders and impaired interoception which is the process integrating internal bodily signals [10–12]. With this regard, several cognitive theories propose that impairments or alterations in interoception may contribute to the development of functional or vicarious symptoms [13]. In addition, epidemiological studies suggest a relation between somatic symptom disorders, defined as the presence of one distressing somatic symptom during at least 6 months, and health anxiety [14].

Bridging a gap between psychosomatic medicine and vicarious symptoms in SPs is crucial for gaining a better understanding of the impact of SPs’ experiences on OSCEs functioning. In the context, we hypothesized that SPs, particularly those with impaired interoception, may be at risk of developing physical symptoms in relation to their role during an OSCE exam. For this study, we sought to (1) assess the prevalence and intensity of new VSs in SPs participating to OSCE exams, and (2) identify risk factors for these VSs focusing on their relationship with interoceptive processes and health anxiety.

## Materials and methods

### Population

The study was carried out on SPs taking part in Medicine Sorbonne University’s OSCEs (Paris, France) during three sessions (OSCE exam for 5th-year medical students). SPs had to be older than 18 years old and to speak fluent French, with no history of hospitalization in the past three months and no family member participating to the OSCE. Of note, a known medical diagnosis and/or an ongoing pharmacological treatment was not an exclusion criterion.

### Design

We conducted a single-center prospective cohort study with three time-points. At T1, two weeks before the OSCEs, all SPs received a proposition by mail to participate to the study. SPs were mostly enrolled among speech therapy students, orthoptics students, and medical educators. If accepted, participants provided socio-demographic information and completed three validated scales assessing health anxiety and interoceptive awareness on online survey as described below. At T2, after the end of the OSCEs day, participants filled out a personalized survey after completing the examination to evaluate the physical symptoms they experienced during the day of the test. Finally, at T3, one week after the OSCEs day, participants completed another personalized online survey to assess physical symptoms reported over the course of the week following the examination.

### Definition and report of vicarious symptoms

VSs were defined as sensations or physical symptoms experienced by SPs related to the organ or disease involved in the OSCE station (i.e., abdominal pain for a situation with an abdominal aortic aneurysm). In addition, SPs were asked to report only the symptoms that they thought to be linked to their role during OSCE exam and sensations or physical symptoms that were not present before the exams. Clinical domains involved covered the field of medical studies (gastro-intestinal, psychiatric, vascular, urinary, cardiovascular, musculoskeletal, respiratory, ENT, neurological, gynecological, endocrine and ophthalmological systems).

VSs were screened at T2 and T3 through 3 questions: two were customized according to the theme of the clinical situation and one was “open” (i.e., any new sensations or physical symptoms related to the role you played). The 2 specific questions were written by two physicians specialized in the field relevant to the station. Intensity of VS were ranged from 0 to 100 using a numerical rate scale. The questions were standardized to minimize the risk of differences between stations.

### Interoceptive awareness

Interoceptive awareness was assessed using the multidimensional assessment of interoceptive awareness version 2 (MAIA-2) [15] and the three-domain interoceptive sensations questionnaire (THISQ) [16]. MAIA-2 is a 32-items and 8-factors scale. These 8 factors are labeled “noticing”, “not-distracting”, “not-worrying”, “attention regulation”, “emotional awareness”, “self-regulation”, “body listening”, and “trust”. “Non-distracting” indicates the tendency to ignore or distract oneself from sensations of pain or discomfort. “Not-worrying” indicates emotional distress or worry with sensations of pain or discomfort. Cronbach’s alpha, measuring internal consistency, was between 0.64 and 0.83. THISQ is a 18-items, 3-factors scale with three categories labeled “cardiorespiratory activation”, “cardiorespiratory deactivation”, and “gastroesophageal sensations” with a Cronbach’s alpha of 0.35 to 0.39.

### Health anxiety

Illness attitudes scale (IAS) was used to assess for health anxiety [17]. This self-reported questionnaire consists of 27 items, which are grouped into 9 distinct domains: worry about illness, concern about pain, health habits, hypochondriacal beliefs, thanatophobia (fear of death), disease phobia, bodily preoccupations, treatment experience, and effects of symptoms. Participants respond to each item using a 5-point Likert scale, ranging from 0 (“no concern”) to 4 (“extreme concern”). For this scale, Cronbach’s alpha varied from 0.52 to 0.81.

### Statistical analysis and definitions

Patients who responded neither at T2 and T3 were excluded from the analysis.

Qualitative data were described as number and percentage. Quantitative data were described as median and interquartile range. Data were compared with Wilcoxon Mann-Whitney and Fisher’s exact tests.

The variable “vicarious symptoms” was analyzed as a binary variable (yes/no). We considered SPs “with vicarious symptoms” when they declared one or more symptoms either at T2 and/or T3 and “without vicarious symptoms” when no symptoms were reported at T2 and T3. To identify factors associated with VSs, we performed a multinomial logistic regression using complete-case analyses, which included variables with *P* < 0.20 on univariate analysis among the following variables: participation year, age, gender, marital status, professional situation, socio-professional category, smoker, level of education, similar situation as the OSCE experienced personally or in the entourage, and the three scores described above. In addition, we performed a univariate analysis to explore correlations between VSs and IAS, THISQ, and subscores.

Finally, we used the Pearson correlation method to test the correlation between the intensity of symptoms and the scores.

We used two-tailed tests and considered *p* < 0.05 to be significant. Statistical analyses were conducted using Stata v15.1 (StataCorp; https://www.stata.com/).

We used STROBE guidelines to report the results.

## Results

### Population characteristics

**Fig. 1:**
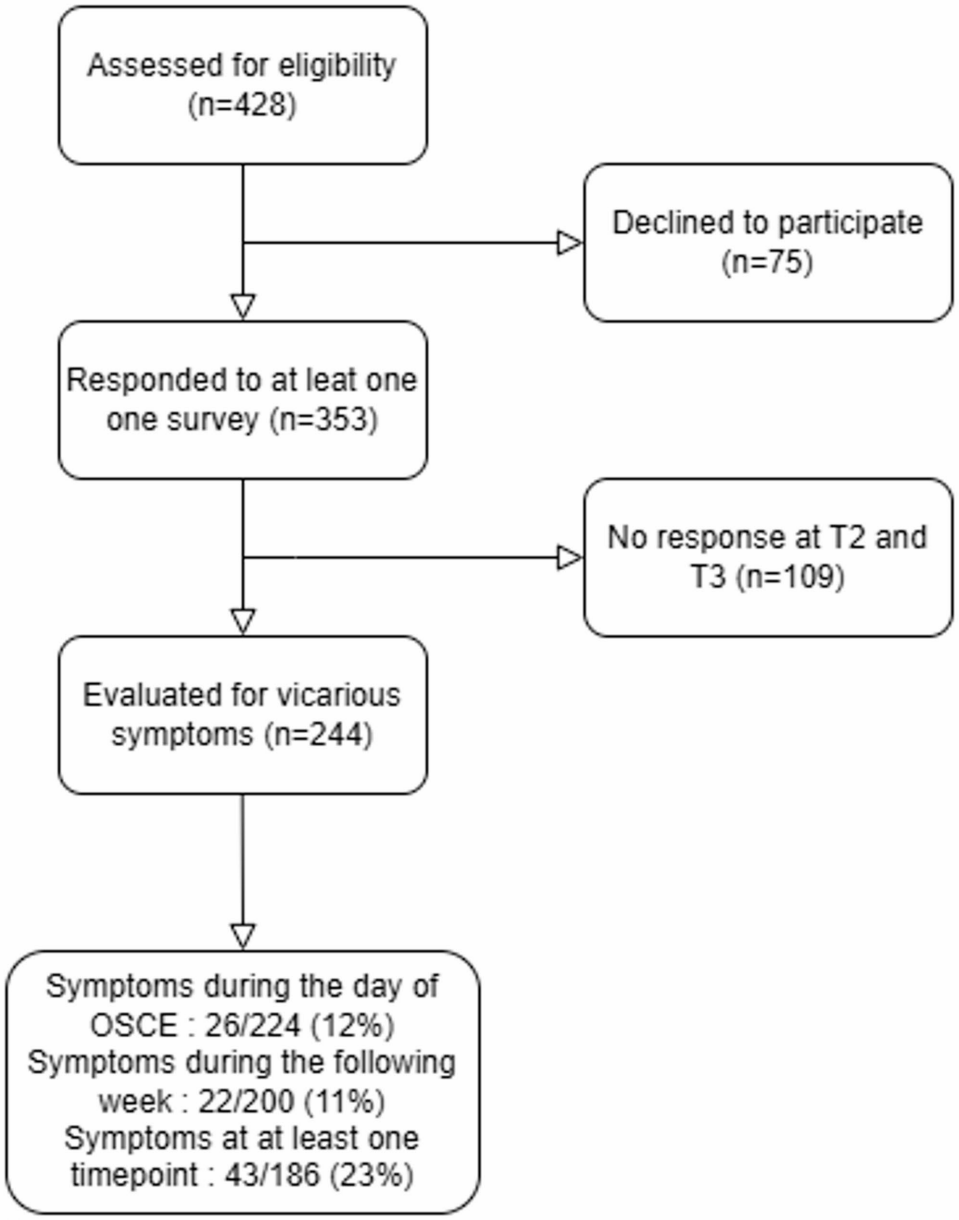
Study flowchart

We collected data from 353 different SPs of which 244 (69%) were analyzed. Figure 1 shows the study flowchart. As described in Table 1, SPs were mostly females (182/205, 89%), students (160/204, 78%) and healthcare workers (speech therapy students, orthoptics students, and medical educators: 166/204, 81%). Compared to healthcare workers, non-healthcare workers were older, more frequently in couples and participated to the OSCE in March 2024, and less frequently students (data not shown). Healthcare worker had higher THISQ total scores (median 61 versus 57, *p* = 0.04). By contrast, we found no significant difference in IAS, and MAIA-2 scores between healthcare workers and non-healthcare workers.

**Table 1:** Socio-demographic and baseline characteristics

	*N* = 244
OSCE session	
June 2023	137/244 (56)
March 2024	47/244 (19)
June 2024	60/244 (25)
Age in year	24 (22–32)
Gender	
Female	182/205 (89)
Male	23/205 (11)
In couple	96/204 (47)
Student	160/204 (78)
Healthcare workers or healthcare students	166/204 (81)
Smoker	26/206 (13)
Education after high school diploma	177/206 (86)
Similar situation/disease experienced personally	48/224 (21)
Similar situation/disease experienced in the entourage	66/224 (29)
IAS total score	40 (27–50)
THISQ total score	61 (53–68)
MAIA-2 total score	2.9 (2.6–3.1)

Qualitative data are n/N (%) and quantitative data are median (interquartile range)

*Abbreviations* *IAS* Illness attitudes scale, *MAIA-2* multidimensional assessment of interoceptive awareness version 2, *THISQ* three-domain interoceptive sensations questionnaire

#### Vicarious symptoms associated with OSCE

Among participants, 26/224 (12%) experienced new VSs the day of the OSCE with a median intensity of 30 (interquartile range 20–50) at T2.

In the week following the OSCEs, 22/200 SPs (11%) experienced unusual VSs while the intensity of their symptoms was evaluated at 45 (30–60). Of note, only 3 subjects consulted a doctor for these symptoms.

Among the 180 SPs who responded to both T2 and T3, VSs were reported for 37 participants at T2 and/or T3 (20%). Of note, only 5/180 (3%) participants reported symptoms both the day of the exam and the following week.

#### Characteristics associated with vicarious symptoms

Among SPs’ characteristics, the experience of a similar situation/disease experienced personally (odds ratio [OR] 2.62, 95% confidence interval [95%CI] 1.22–5.63, *p* = 0.01) and the IAS score (OR 1.03 for each additional point, 95%CI 1.01–1.05, *p* = 0.02) were associated with VSs. As described in Table 2, we found similar results using a multivariate analysis (adjusted OR 3.07, 95%CI 1.33–7.07, *p* = 0.008 and 1.03, 95%CI 1.01–1.05, *p* = 0.03, respectively).

**Table 2:** Univariate and multivariate analysis of factors associated with the presence of vicarious symptoms

	Univariate analysis		Multivariate analysis	
	OR (95%CI)	p-values	aOR (95%CI)	p-values
Healthcare workers	1.03 (0.42–2.50)	0.94		
Age	1.00 (0.97–1.03)	0.98		
Student	0.82 (0.35–1.88)	0.64		
In couple	1.25 (0.62–2.54)	0.53		
Education after high school diploma	0.59 (0.23–1.51)	0.28		
Smoker	1.46 (0.48–4.49)	0.51		
Disease experienced personally	2.62 (1.22–5.63)	0.01	3.07 (1.33–7.07)	0.008
Disease experienced in the entourage	1.70 (0.82–3.51)	0.15		
Gender				
Male	Reference			
Female	2.35 (0.51–10.83)	0.23		
OSCE session				
June 2023	Reference			
March 2024	0.87 (0.38–1.99)	0.07		
June 2024	0.09 (0.01–0.69)			
IAS total score	1.03 (1.01–1.05)	0.02	1.03 (1.01–1.05)	0.03
THISQ total score	1.00 (0.98–1.04)	0.53		
MAIA-2 total score	0.99 (0.44–2.27)	0.99		

*Abbreviations* *OR* odds ratio, *aOR* adjusted odds ratio, *IAS* Illness attitudes scale, *MAIA-2* multidimensional assessment of interoceptive awareness version 2, *THISQ* three-domain interoceptive sensations questionnaire

Using univariate analysis, we found that 1 IAS subitems, “bodily preoccupations” (OR 1.21, 95%CI 1.04–1.41, p = 0.01), was associated with the presence of VSs. In addition, we observed a significant association between the MAIA-2 subitem “not-worrying”, which reflects the tendency not to worry or experience emotional distress in response to sensations of pain or discomfort, and the absence of VSs (OR 0.52, 95%CI 0.31–0.87, p = 0.01, Supplemental Table 1).

#### Association between interoception, health anxiety and intensity of vicarious symptoms

Using Pearson correlation test, we found no significant correlation between IAS, THISQ, and MAIA-2 scores and VSs intensity at T2 and T3 among SPs that reported VSs (Pearson correlation coefficient ranging from -0.07 to 0.27, Supplemental Table 2).

#### Impact of vicarious symptoms on discomfort and future OSCE participation

Participants who experienced VSs during the OSCE day reported significantly more frequently discomfort during the OSCE role-playing (8/18 [31%] versus 22/198 [11%], *p* = 0.01). Just after the OSCE session, 43/224 subjects (19%) did not imagine participating the following year. After one week, 45/200 subjects (22%) no longer wished to repeat OSCEs the following year. We found no correlation between VSs and volunteering for participating in the following year.

## Discussion

In our study, approximately one in five SPs participating in the OSCEs reported new VSs, either on the day of the assessments or during the following week. The occurrence of these symptoms was linked to higher health anxiety scores and prior personal experience with the simulated situation or illness. Furthermore, approximately 20% of SPs no longer wish to participate in future OSCEs tests findings highlighting an important challenge.

Previous studies have described physical symptoms related to their role among SPs. However, most studies focused on non-specific symptoms such as stress [3, 4] or discomfort [18]. Some studies explored very specific situations such as simulation of an HIV-positive patient, gynecological examination or OSCE with SPs selected because they were considered “clinically obese” [3, 5–7, 19, 20] and were limited by the low number of participants. The situation we analyzed could be more in line with “real life” OSCE exam. Furthermore, our study provides new data on the correlation between SP characteristics and the appearance of VSs.

The occurrence of VSs was significantly associated with the SPs’ prior personal experience of a similar situation or disease to the one simulated during the OSCEs. This finding suggests that such symptoms could, at least partially, be mitigated if SPs were selected without a history of personal exposure to the condition or scenario being simulated. By reducing the emotional resonance or identification with the simulated role, the risk of experiencing VSs may decrease. Moreover, implementing structured debriefing sessions after the simulations has been proposed as a potential strategy to alleviate these symptoms [7]. Debriefing allows participants to process their emotional responses, address any lingering distress, and better separate the simulated experience from their personal reality. Such interventions could play a key role in minimizing the psychological and physical impact of high-intensity simulations on SPs.

We observed an association between VSs and higher IAS scores. Especially, we observed a correlation between higher score concerning the “bodily preoccupations” subscale and VSs appearance. Kellner proposed that subscales of the IAS score may assess psychopathology associated with hypochondriasis [21]. For the appearance of VSs, we can hypothesize that the underlying mechanism is PSs being overly aware of sensations experienced during simulation and interpreting them as worrisome. By contrast, other subscales exploring other mechanisms may not be in play (i.e., as the subscale “concerns about pain” which focuses on the interpretation of physical pain as a sign of an underlying disease). This raises the question about prevention methods focusing on this mechanism. Interestingly, this subscale has been proposed as screening tool for hypochondriasis because of satisfactory internal consistency, high sensitivity, high specificity and the gain in time compared to the complete IAS score [22]. Moreover, 35% of the SPs had a baseline IAS score ≥ 47, as proposed for a cut-off for the diagnosis of health anxiety (with a sensitivity and specificity of 95%) [23]. The prevalence of hypochondria in the general population is highly variable due to the variability of the scales used [24]. In addition, several studies have suggested that female gender [25, 26], young age, and being a student increase the risk of health-related anxiety [25]. These risk factors correspond to the characteristics of SPs enrolled in this study. Recently, a meta-analysis has reported that 28% of Chinese health science students presented hypochondriac symptoms [27]. Of note, it is necessary to differentiate between the presence of health anxiety symptoms, as measured by the IAS scale in our study, and health anxiety disease which can be diagnosed only if these symptoms persist for more than 6 months [28]. Similarly, appearance of somatic symptoms is to differentiate from somatic symptom disorder which requires the presence of at least one distressing somatic symptom during at least 6 months [14]. We cannot exclude that their preparation for the SP role may have transiently increased health-related anxiety. A future study should evaluate these variables well in advance of the preparation of OSCEs. Interestingly, the subscale “not worrying” of the MAIA-2 scale was also correlated to VSs. It is noticeable that questions of this subscale, such as “I can notice an unpleasant body sensation without worrying about it”, evaluate similar aspects as the subscale “bodily preoccupations” of the IAS scale.

Contrary to our hypothesis, we found no correlation between global MAIA-2 and THISQ scores, evaluating interoception, and VSs. Interoception disorders have been described in somatic symptoms disorders which present similar aspects to VSs appearance [12]. However, our results suggests that the appearance of VSs is more linked to illness anxiety than interoceptive disorders. MAIA-2 and THISQ scores explore the integration of bodily sensations, as discussed previously, but also other aspects less related to the situation of SPs such as integration of internal physical sensations and their regulation. In addition, these scores are not optimal to assess the integration of external factors such as being asked to simulate chest pain.

About 20% of SP reported that they did not wish to participate again the following year, mainly because participation was mandatory as part of their studies. However, we did not identify any significant association between this reluctance and the occurrence of case-related VSs. Future studies with larger samples could further investigate potential factors contributing to this decision, such as emotional distress, perceived difficulty of the role, or dissatisfaction with the experience. This reluctance to return poses significant organizational challenges, particularly in ensuring the consistent execution of OSCEs. It highlights the need to explore alternative recruitment strategies, such as diversifying the pool of SPs or implementing measures to enhance their satisfaction and well-being, including more robust training, support, and post-simulation follow-up.

Several points warrant consideration regarding the OSCEs and their potential impact on participants. First, the issue of the number of repetitions of each station per day band/or per participant, is rarely addressed. Yet it could play a significant role in the psychological stress or potential trauma induced by the examination as suggested in a qualitative study on 35 SPs [5]. It is possible that repeated exposure to the same clinical scenario may amplify feelings of anxiety or distress, particularly if these repetitions are perceived as a form of failure or inadequacy. One possible way to explore this would be by varying the number of repetitions—doubling it, for example—to assess a potential dose-response effect and its impact on stress levels. Second, the discussion on the preparation leading up to the OSCE is often underexplored. The way participants prepare for the examination, both cognitively and emotionally, may have a profound effect on their performance and stress responses. This area presents an opportunity to enhance OSCE preparation by targeting “at-risk” participants with tailored interventions or techniques designed to alleviate anxiety or bolster confidence. Identifying those who may struggle more with specific aspects of the exam and offering personalized support could reduce stress and improve overall performance, potentially reducing the negative impact of the examination on their well-being.

Our study has several limitations. First, we observed a significant amount of missing data. It is possible that the profile of those lost to follow-up differs from that of the respondents. This limitation is inherent to the study design, which involved data collection at three different time points. Additionally, we believe that assessing health anxiety and interoceptive awareness after the OSCEs may have introduced bias, further complicating the analysis. Second, our sample was highly homogeneous, consisting primarily of young female health students due to the recruitment methods. Inclusion criteria for SPs may differ from one center to another. Future studies should include more socio-demographically diverse populations, reflecting the broader range of individuals recruited as SPs in different contexts. Third, we did not implement an intervention to address VSs due to ethical constraints, which limited our ability to evaluate potential strategies for symptom management. Fourth, we were unable to perform subgroup analyses based on the specific OSCE scenario, as the high number of different situations (*n* = 20) compared to the total number of SPs enrolled (*n* = 244) made such analyses statistically unfeasible. Future research could address this by focusing on fewer, more comparable scenarios or enrolling larger cohorts. Fifth, we haven’t assessed the long-term effects. These effects have been rarely reported [3] and their assessment would have been challenging regarding the missing data after one week. Finally, we haven’t explored the medical students’ perspective. Their scores and satisfaction may be affected by VSs appearance among SPs.

This study offers new opportunities for dialogue between cognitive sciences and medical education. Integrating IAS into the selection process for SPs could improve the precision and consistency of patient profiles, ensuring that they align more closely with the needs of the simulation. Considering individual characteristics during patient selection is essential to ensure that the data collected is both reliable and representative. Future research should focus on developing strategies to prevent and manage case-related VSs, especially in SPs with high IAS scores. This could lead to better support systems and improved outcomes for both the patients and the medical education process.

## Conclusion

VSs affect approximately one in five SPs. These symptoms are significantly associated with higher scores on the IAS and a prior personal experience with a similar illness. This underscores the potential influence of both health-related anxiety and personal history on the physical responses of SPs during simulations.

## Supplementary Information

Supplementary Material 1.

## Data Availability

Data are available upon reasonable request to the corresponding author.

## Abbreviations

aOR: Adjusted odds ratio
IAS: Illness attitudes scale
MAIA-2: Multidimensional assessment of interoceptive awareness version 2
OR: Odds ratio
OSCE: Objective structured clinical examination
SP: Standardized patient
THISQ: Three-domain interoceptive sensations auestionnaire
VS: Vicarious symptom
