# Supplementary material for: When simulation becomes physical: vicarious symptoms in standardized patients during osces
Source: BMC Med Educ. 2025 Oct 28;25:1509. doi: 10.1186/s12909-025-07950-w (PMC12570795; doi:10.1186/s12909-025-07950-w)
Supplement: Supplementary file 1 — Supplementary Material 1. [file 12909_2025_7950_MOESM1_ESM.docx]

**Supplemental Table 1**. Univariate analysis of the association between IAS, THISQ and MAIA-2 subscores with the presence of vicarious symptoms.

|  | Univariate analysis | |
| --- | --- | --- |
|  | OR (95%CI) | p-values |
| **IAS subscores**  Worry about illness  Concerns about pain  Health habits  Hypochondriacal beliefs  Thanatophobia  Disease phobia  Bodily preoccupations  Treatment experience  Effects of symptoms | 1.08 (0.96-1.22)  1.08 (0.94-1.24)  1.00 (0.87-1.16)  1.17 (0.99-1.37)  1.06 (0.95-1.17)  1.10 (0.99-1.23)  **1.21 (1.04-1.41)**  1.15 (0.99-1.35)  1.13 (0.98-1.29) | 0.18  0.26  0.91  0.06  0.28  0.07  **0.01**  0.06  0.08 |
| **THISQ subscores**  Cardiorespiratory activation  Cardiorespiratory deactivation  Gastroesophageal sensations | 1.05 (0.97-1.14)  0.98 (0.92-1.05)  1.04 (0.96-1.11) | 0.23  0.58  0.30 |
| **MAIA-2 subscores**  Noticing  Not-distracting  Not-worrying  Attention regulation  Emotional awareness  Self-regulation  Body listening  Trusting  Unweighted score | 1.61 (0.94-2.77)  0.86 (0.50-1.49)  **0.52 (0.31-0.87)**  1.01 (0.62-1.64)  1.12 (0.71-1.75)  1.02 (0.66-1.58)  1.34 (0.92-1.95)  0.95 (0.65-1.38)  1.01 (0.92-1.11) | 0.08  0.59  **0.01**  0.96  0.62  0.92  0.12  0.78  0.83 |

Abbreviations: OR, odds ratio; IAS, Illness attitudes scale; MAIA-2, multidimensional assessment of interoceptive awareness version 2; THISQ, three-domain interoceptive sensations questionnaire

**Supplemental Table 2.** Correlation between VSs intensity at T2 and T3 and IAS, THISQ, and MAIA-2 scores.

|  | Pearson correlation coefficient | p-values |
| --- | --- | --- |
| Intensity at T2  IAS score  THISQ score  MAIA-2 score | 0.12  0.02  -0.03 | 0.57  0.91  0.90 |
| Intensity at T3  IAS score  THISQ score  MAIA-2 score | -0.07  0.27  0.19 | 0.76  0.22  0.39 |

Abbreviations: IAS, Illness attitudes scale; MAIA-2, multidimensional assessment of interoceptive awareness version 2; THISQ, three-domain interoceptive sensations questionnaire
